# Antibacterial Effects of *Cissus welwitschii* and *Triumfetta welwitschii* Extracts against *Escherichia coli* and *Bacillus cereus*

**DOI:** 10.1155/2015/162028

**Published:** 2015-11-05

**Authors:** Batanai Moyo, Stanley Mukanganyama

**Affiliations:** Biomolecular Interactions Analyses Laboratory, Department of Biochemistry, University of Zimbabwe, P.O. Box MP167, Mount Pleasant, Harare, Zimbabwe

## Abstract

Antibiotic resistance has increased sharply, while the pace for the development of new antimicrobials has slowed down. Plants provide an alternative source for new drugs. This study aimed to screen extracts from *Cissus welwitschii* and *Triumfetta welwitschii* for antibacterial activity against *Escherichia coli* and *Bacillus cereus*. The tests conducted included a susceptibility determination test, analysis of the effect of *T. welwitschii* on cell wall integrity, and transport across the membrane. It was found that the *T. welwitschii* methanol extracts were more effective than the water extracts and had the lowest minimum inhibitory concentration and minimum bactericidal concentration at 0.125 mg/mL and 0.5 mg/mL, respectively, against *E. coli* and *B. cereus*. The *C. welwitschii* extract caused the most drug accumulation in *E. coli*. In *B. cereus*, no significant drug accumulation was observed. Nucleic acid leakage in *B. cereus* and *E. coli* and protein leakage in *E. coli* were observed after exposure to the *T. welwitschii* extract. The extracts from *T. welwitschii* had greater antibacterial activity than the extracts from *C. welwitschii*. *T. welwitschii* may be a potential source of lead compounds for that could be developed into antibacterial agents.

## 1. Introduction

Antibiotic resistance has become a major problem worldwide in recent years. The unnecessary prescription of antibiotics to treat viral infections, incorrect prescriptions due to a lack of culturing to determine the exact cause of infection, patients not completing their antibiotic treatments, and the excessive use of antibiotics in stock feed are some of the causes of the increased selective pressures placed on bacteria by antibiotics, leading to resistance [1]. There has been a decrease in the development of antimicrobial agents by pharmaceutical companies [2] as their development is not as profitable as developing drugs that treat chronic and lifestyle diseases [3]. In the last four decades, only three new classes of antibiotics have been discovered which are lipopeptides, oxazolidinones, and streptogramins [4]. As most of the old and cheap antibiotics are no longer effective, the use of second or third line drugs has become necessary, and these may have side effects [5]. When patients are infected with organisms resistant to all available antimicrobials, surgery is required to remove the nidus of infection, increasing the risk of death [6]. Drug resistance is of particular concern among patients with compromised immunity [7]. Infectious diseases account for half of all deaths in tropical countries [8] and 90% of infections are caused by bacteria [9].

Bacteria have the ability to acquire resistance mechanisms such as genes encoding enzymes such as *β*-lactamases, genes that alter the bacterial cell wall resulting in no binding site for the antimicrobial agent and efflux pumps through conjugation, translation, and transduction [10]. Efflux pumps are transport proteins involved in the extrusion of toxic substrates (including almost all classes of clinically relevant antibiotics) [9]. Efflux pumps either can be selective or can be multidrug efflux pumps, and the extrusion of antimicrobial agents via these efflux pumps is a major component of resistance [11]. Cells can use proton driven antiporters and/or ATP driven (ATP-binding cassette) transporters to expel drugs [12]. Efflux pump inhibitors can be used to restrict the efflux of antibiotics from bacterial cells, meaning that resistance to antibiotics such as ciprofloxacin can be reversed and drug resistance can be inhibited [13]. Examples of commonly used efflux pump inhibitors are verapamil and MC-207,110 [12]. As a result of antibiotic resistance, new antimicrobials that will either replace or be used with antibiotics are required.

In African countries, traditional medicines are used by approximately 80% of the population, while usage of alternative medicines in developed countries is increasing [14]. Traditional medicines are extensively incorporated into the public health system of some countries [15]. To date, studies have confirmed that some plant extracts can be used to treat infectious diseases caused by bacteria as they have antibacterial properties [16–18]. Studies have been conducted to determine the effects of plant extracts on the activity of antibiotics against resistant bacteria [7, 9]. Plant extracts have been used to inhibit microbial growth in food and drinks [19].

Interest in herbal medicines has increased in recent years due to the fact that they are cheap, readily available, and effective, as well as the high cost of industrialized medicines, lack of access to healthcare, and the side effects caused by taking synthetic medicines [20]. Natural products offer a large diversity of chemical structures which often serve as lead molecules whose activities can be enhanced by manipulation through combinations with chemicals and by synthetic chemistry [21, 22]. Novel compounds from plants may not have the problem of antimicrobial resistance [21]. Natural plant extracts have been found to be an important source of secondary metabolites such as tannins, terpenoids, alkaloids, and flavonoids, which have been found* in vitro* to have antimicrobial properties [22]. Some plant extracts have also been classified as resistance modifiers because they can enhance antibiotic activity or reverse antibiotic resistance [22]. In Zimbabwe, plants have been used for centuries to treat various ailments. Examples are* Vernonia adoensis* leaves for the treatment of tuberculosis and* Salons delagoense* leaves and fruits for the treatment of scabies in children [23].* Aloe excelsa*,* Cymbopogon nardus*, pepper, coconut milk, and coconut oil are some plants used for the preparation of ointments, lotions, and creams [24].

The aim of this study was to investigate the antibacterial properties of the aqueous and methanol extracts of* Cissus welwitschii* and* Triumfetta welwitschii* var.* welwitschii*. The genus* Cissus* contains 350 species and is found in tropical and subtropical locations [25].* Cissus* is a member of the grape family Vitaceae [25].* C. welwitschii* is found in tropical Africa, often on granite outcrops and termite mounds and in forests, semievergreen bushland, and woodland [26]. The plant is a vigorous grower, that is, 1.8–9 m long or shrubby, and has aerial roots [26].* C. welwitschii* has hairless, cylindrical stems that are often spotted black and granular [26]. The plant has simple leaves and tendril, and is closely related to* C. fragilis* [27].* C. welwitschii* is used traditionally in Zimbabwe for the treatment of cancer.

*Triumfetta welwitschii* var.* welwitschii* is a perennial herb that grows annual stems from a woody rootstock and usually flowers before the leaves develop [28]. The plant is a conspicuous species of burnt roadsides, grassland, and woodland [28]. Three varieties of* T. welwitschii* exist, namely,* welwitschii*,* descampsii,* and* hirsuta* [28]. In Zimbabwe,* T. welwitschii* is used to treat diarrhoea [29], suggesting that it has antibacterial effects. Microorganisms contribute to food spoilage and in turn diarrhoea, so treatment of diarrhoea using the* T. welwitschii* plant extract may indicate that the plant has antibacterial activity. In South Africa, a decoction of the tuber is mixed with milk and drunk as a fever remedy [30] suggesting that it has antipyretic effects.

*Escherichia coli* and* Bacillus cereus* were the two bacteria used in this study. Nonpathogenic strains of* E. coli* live in the human colon. However, several strains are important foodborne pathogens. Pathogenic strains include* E. coli* O157:H7 which was first discovered in 1982 [1]. This strain is found in meat products, unpasteurised fruit juices, fruits, vegetables, and untreated water and is known to cause haemolytic uremic syndrome [1].* E. coli* O55, O111, and O127 have been associated with infant diarrhoea, while other strains have been associated with nosocomial infections in the skin, urinary tract, and surgical wounds [1].* E. coli* strains resistant to drugs including penicillins and cephalosporins have been discovered [1].

*B. cereus* causes food poisoning and is especially problematic in starchy foods [1]. The emetic toxin produced by* B. cereus* has been associated with improperly stored boiled and fried rice (causes nausea and vomiting) while the diarrheal type is associated with a wider range of foods [1]. As* B. cereus* and* E. coli* cause very serious illnesses, new drugs are needed that can adequately manage the infections that they cause.

The plants used in traditional medicine are potential sources of antibacterial agents. Many plants used traditionally achieve good results, so studying these plants may give rise to new potent antimicrobials with different mechanisms of action. The results of this study can be used to either validate or invalidate the use of* T. welwitschii* and* C. welwitschii* in traditional medicine.

## 2. Materials and Methods

### 2.1. Chemicals

All chemicals and antibiotics used were purchased from Sigma-Aldrich, Steinheim, Germany, and were of analytical grade. The major chemicals and antibiotics used were methanol, for extractions, reserpine, an efflux pump inhibitor, rhodamine 6G, a probe compound for the drug efflux assay, propidium iodide dye, used to stain nucleic acids, Bradford's Reagent for protein determination using the Bradford's assay, and the three antibiotics used in the study: ampicillin, kanamycin, and norfloxacin.

### 2.2. Plant Material

*Cissus welwitschii* and* Triumfetta welwitschii* were collected from Centenary (16.8°S, 31.1167°E, and 1 156 m above sea level), Mashonaland Central Province, Zimbabwe. The plants were identified and authenticated by Mr. Christopher Chapano, a botanist with the National Botanical and Herbarium Garden (Harare, Zimbabwe).

### 2.3. Preparation of Extracts

The dried leaves and roots of each plant were ground separately in a blender (Philips Co., Shanghai, China). To 10 g of plant material, either 100 mL of methanol or 200 mL of distilled water was added. Two hundred millilitres of distilled water was used as the mixture became viscous during stirring. The mixtures were stirred using a magnetic stirrer for 20 minutes. The methanol extracts were filtered using No. 1 Whatman filter paper, while the water extracts were filtered using mutton cloth. The filtrate was evaporated to dryness, collected, and stored at room temperature.

### 2.4. Bacteria

*Escherichia coli* ATCC 11229 and* Bacillus cereus* ATCC 11778 were used in this study. The bacteria were grown from glycerol stocks stored at −33°C. On nutrient agar plates, 25 *µ*L of bacteria was plated and grown overnight at 37°C in an incubator (Lab Design Engineering (Pty) Ltd., Maraisburg, South Africa). From the plates, 1 colony was inoculated into nutrient broth and grown overnight at 37°C with shaking at 160 revolutions per minutes (rpm) (Lab Companion, Jeio Tech, South Korea). The plates and bacterial culture tubes were stored at 4°C. Fresh stocks were prepared for each assay.

### 2.5. Determination of Antibacterial Activity Using the Agar Disk Diffusion Method

Molten nutrient agar was inoculated with bacteria to a final concentration of 1 × 10^6^ cfu/mL. The agar was poured into petri dish plates and allowed to solidify. Stock solutions of* C. welwitschii*,* T. welwitschii,* and the antibiotic ampicillin were prepared to a concentration of 25 mg/mL by dissolving the plant extracts in the extracting solvent and ampicillin in distilled water. Filter paper disks measuring 6 mm in diameter were prepared. To each disk, 20 *µ*L of extract or antibiotic was added. The samples were prepared in quadruplicate. The disks were allowed to dry before being placed onto the plates impregnated with bacteria. The plates were stored at 4°C for two hours to allow the extracts and antibiotics to diffuse into the agar. The plates were incubated overnight at 37°C. The diameters of the zones of inhibition were measured in millimetres.

### 2.6. Minimum Inhibitory Concentration (MIC) and Minimum Bactericidal Concentration (MBC)

The MICs of the plant extracts and antibiotics against* B. cereus* and* E. coli* were determined using the broth dilution method. The assay was based on the method described by Eloff [31]. One in two serial dilutions of the extracts and antibiotics were prepared. For the plant extracts and ampicillin, the concentrations used ranged from 0.008 mg/mL to 4 mg/mL, while, for kanamycin and norfloxacin, the concentrations used ranged from 1 *µ*g/mL to 1 mg/mL. One hundred microliters of the extracts and antibiotics was added to wells in microwell plates. To each well, 100 *µ*L of bacteria was added to achieve a final concentration of 1 × 10^6^ cfu/mL. Wells containing 200 *µ*L of broth only, 100 *µ*L of the extracts or antibiotics and 100 *µ*L of broth, and 100 *µ*L of broth and 100 *µ*L of bacteria were used as the controls. The plates were incubated at 37°C with shaking at 30 rpm overnight.

After incubation, the absorbance of the wells was measured at 600 nm using a microplate spectrophotometer (SpectraMaxPlus, Molecular Devices, Sunnyvale, USA). To each well, 25 *µ*L of 3-(4,5-dimethylthiazol-2-yl)-2,5-diphenyltetrazolium bromide (MTT) was then added. MTT turns dark blue (from yellow) in the presence of living cells [32]. The plates were incubated for 1 hour at 37°C at 30 rpm. The absorbance was measured at 570 nm.

To determine the MBC, one loopful of the bacteria in the wells that contained the MIC was streaked, in duplicate, onto nutrient agar plates without antibiotics. The wells that contained extracts at one and two concentrations higher and one concentration lower than the MIC were also plated. The plates were incubated for 24 hours at 37°C.

### 2.7. Effects of Plant Extracts on Drug Accumulation

The drug accumulation assay was used to determine the effects of the plant extracts on drug accumulation in* E. coli* and* B. cereus*. The method described by Chitemerere and Mukanganyama [23] was used with some modifications. The bacteria were grown overnight at 37°C in two separate flasks containing 400 mL nutrient broth (120 rpm). The bacteria were centrifuged at 4000 rpm for 10 minutes (MSE Minor 35, England). The supernatant was discarded. The pellet was washed twice in phosphate buffered saline (PBS) (pH 7.2). The cells were centrifuged at 4000 rpm for 5 minutes in a preweighed tube. The supernatant was discarded and the pellet was weighed using a Kern EG balance (Kern & Sohn, Germany). PBS containing 10 mM sodium azide (NaN_3_) was added to the tube to achieve a concentration of cells of 40 mg/mL. The tube was gently inverted to disperse the cells within the PBS. Rhodamine 6G (R6G) to a final concentration of 10 *µ*M was immediately added and cells were incubated at 90 rpm for 1 hour.

The cells were divided into two tubes, A and B, in the ratio of 1 : 2 but both with the same concentration of cells. Both tubes were centrifuged at 4000 rpm for 5 min. For tube A, the supernatant was discarded, and PBS alone was added to achieve a final concentration of cells of 40 mg/mL. For tube B, the supernatant was discarded and the pellet was resuspended in PBS containing 1 M glucose. The contents of tube B were then divided into five tubes, B_1_ to B_5_. Reserpine was added to tube B_1_ to a final concentration of 60 ng/mL. Tube B_2_ served as a control with glucose alone. Plant extracts were added to tubes B_3_ to B_5_ to achieve final concentrations of 60 ng/mL of plant extract in each tube. Equal concentrations of plant extracts and reserpine were used to allow comparisons to be conducted. All of the tubes were mixed on a vortex mixer (Barnstead/Thermolyne, USA) before being incubated for 30 minutes (90 rpm, 37°C) in a Lab Companion SI-300 incubator.

After incubation, the tubes were centrifuged at 4000 rpm for 10 minutes and the supernatant was discarded. The pellets were resuspended in 0.1 M glycine HCl, pH 3. The glycine HCl was used to lyse the cells. The cells were mixed on a vortex mixer before being incubated at 37°C, 90 rpm overnight. The tubes were centrifuged at 4000 rpm for 10 minutes and the supernatant was collected. The absorbance of R6G was measured at 527 nm. The standards were prepared by diluting R6G in glycine HCl, and the concentrations used ranged from 0 *µ*M to 5 *µ*M.

### 2.8. Determination of the Effect of* T. welwitschii* Extracts on Nucleic Acids Leakage

Propidium iodide is a dye that is capable of binding to nucleic acids. The dye is unable to enter viable cells, making it useful for determining the effects of plant extracts on bacterial membranes.* B. cereus* and* E. coli* cells were suspended in 0.9% saline solution (OD_600_ = 1.5). The cell suspensions were exposed to plant extracts at concentrations of the MIC and double the MIC in duplicate for 10 minutes. The bacteria (1 mL) were centrifuged for 1 minute at 11000 ×g (Centrifuge 5415C, Eppendorf, Berlin, Germany). The pellet was washed with 1 mL 0.9% saline solution. Three microliters of propidium iodide was added to each sample and the solution was mixed. The samples were kept in the dark for 10 min. Fluorescence was measured at excitation and emission wavelengths of 544 nm and 612 nm, respectively, using an *f*_max_ microplate spectrofluorometer (Molecular Devices, Sunnyvale, USA). The controls used were nontreated cells, 3% DMSO, 0.1% SDS, and kanamycin.

### 2.9. Determination of the Effect of* T. welwitschii* Extracts on Protein Leakage

*B. cereus* and* E. coli* cells were suspended in 0.9% saline solution (OD_600_ = 1.5). The cell suspensions were exposed to plant extracts at concentrations of the MIC and double the MIC. The samples were incubated at 37°C with shaking (120 rpm) for 120 min. Five hundred microlitres of cell suspension was centrifuged at 7000 rpm for 2 min. To 50 *µ*L of the supernatant, 950 *µ*L of Coomassie brilliant blue G-250 was added to measure the protein content by Bradford's method. The colour was allowed to develop for 10 min before the absorbance was measured at 595 nm using a spectrophotometer. The controls used were kanamycin, 3% DMSO, 0.1% SDS, and untreated cells. Bovine serum albumin (BSA) was used as a standard to determine protein concentration.

### 2.10. Statistical Analysis

The one-way analysis of variance test (ANOVA) with Dunnett's Multiple Comparison Test was used to analyse the results. The values with a *p* value of 0.05 or less were considered statistically significant. GraphPad Prism 5 for Windows (GraphPad Software Inc., San Diego, California, USA) version 5.03 was used.

## 3. Results

### 3.1. Determination of Antibacterial Activity Using the Disk Diffusion Method

The results of the disk diffusion assay are shown in Table 1. The zone of inhibition refers to the diameter of the zone in which no growth of bacteria was observed minus the diameter of the filter paper disk (6 mm). The root methanol extract from* T. welwitschii* had the largest zones of inhibition against both* B. cereus* and* E. coli* at 6 mm and 4.5 mm, respectively.* T. welwitschii* leaf and root methanol extracts produced larger zones of inhibition against the bacteria than* C. welwitschii* leaf and root methanol extracts. The leaf water extracts of both* C. welwitschii* and* T. welwitschii* produced no zones of inhibition, making them the least effective extracts. Generally the zones of inhibition are larger against* B. cereus* than against* E. coli*. The ampicillin was more effective at inhibiting* E. coli* than* B. cereus* (12 mm and 9 mm, resp.).

### 3.2. Minimum Inhibitory Concentration and Minimum Bactericidal Concentration

The results of the MIC assay, shown in Table 2, show that* C. welwitschii* leaf and root methanol extracts were more effective against* E. coli* than* B. cereus*. The* T. welwitschii* root water and methanol extracts were more effective against* B. cereus* than* E. coli*. The* T. welwitschii* leaf methanol showed significant growth inhibitory effect against both* E. coli* and* B. cereus* with an MIC of 0.125 mg/mL. All 3 antibiotics, particularly kanamycin and norfloxacin, inhibited* B. cereus* and* E. coli* at very low concentrations.* B. cereus* and* E. coli* were inhibited at 0.5 *µ*g/mL and 1 *µ*g/mL, respectively, by kanamycin and 0.25 *µ*g/mL by norfloxacin. Of the plant extracts,* T. welwitschii* leaf methanol extract had the lowest MBC of >0.5 mg/mL against* B. cereus* and* E. coli*.* C. welwitschii* leaf and root methanol extracts and* T. welwitschii* root water extract were the highest at >4 mg/mL. The antibiotics had lower MICs and MBCs against* B. cereus* than* E. coli*, except norfloxacin which had the same MIC for both bacteria. Of note was that at 4 mg/mL of* C. welwitschii* root methanol extract,* E. coli* grew fairly well, but at 2 mg/mL the extract killed the bacteria.

### 3.3. Effects of Plant Extracts on Drug Accumulation

**Figure 1:**
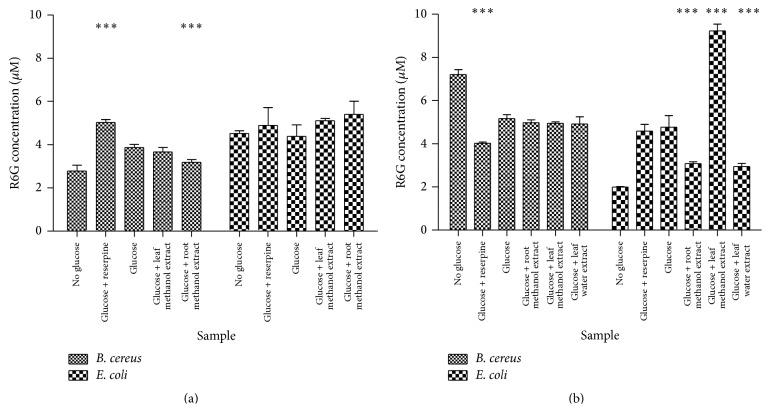
Drug accumulation in* B. cereus* and* E. coli* cells. The graphs show the accumulation of R6G in* B. cereus* and* E. coli* cells after exposure to* T. welwitschii* (a) and* C. welwitschii* (b) extracts. The amount of R6G accumulated is measured in *μ*M. The mean and standard deviation are shown. *n* = 3. The test was conducted three times. The test for significance was carried out by comparing glucose + reserpine/plant extracts to glucose only. ^*∗∗∗*^ *P* < 0.001.

The results of the R6G accumulation assay using* T. welwitschii* extracts are shown in Figure 1(a). Generally more drug accumulation was observed in* E. coli* (>4 *µ*M) than in* B. cereus* (<4 *µ*M). In the presence of the leaf methanol and root methanol extracts 5.10 *µ*M and 5.39 *µ*M R6G, respectively, accumulated in* E. coli*, while in the presence of reserpine 4.89 *µ*M R6G was accumulated. For* E. coli* none of these results were significantly higher than the control: cells exposed to glucose only (4.38 *µ*M). When the* B. cereus* cells were exposed to reserpine, 5.01 *µ*M R6G was accumulated in the cells. There was a significant decrease in R6G accumulation when* B. cereus* cells were exposed to the* T. welwitschii* root methanol extract.

Figure 1(b) shows the accumulation in* B. cereus* and* E. coli* after exposure to* C. welwitschii* extracts. In* E. coli*, the leaf methanol extract caused the accumulation of large amounts of R6G (9.25 *µ*M). The remaining* C. welwitschii* extracts, the root methanol and leaf water extracts, caused much less accumulation at 3.06 *µ*M and 2.93 *µ*M, respectively, than the control cells that were exposed to glucose. In* E. coli*, more R6G accumulated inside the cells treated with glucose than in those treated without glucose (4.65 *µ*M and 1.98 *µ*M, resp.). In* B. cereus*,* C. welwitschii* extracts caused more drug accumulation than that observed in cells treated with reserpine, but the accumulation was not significantly different to cells exposed to glucose only. The root methanol, leaf methanol,and leaf water extracts caused the accumulation of 4.98 *µ*M, 4.95 *µ*M, and 4.92 *µ*M R6G, while reserpine caused the accumulation of 4.03 *µ*M. The cells exposed to glucose had more accumulation of R6G than those without glucose.

### 3.4. Determination of the Effect of* T. welwitschii* Extracts on Nucleic Acids Leakage

**Figure 2:**
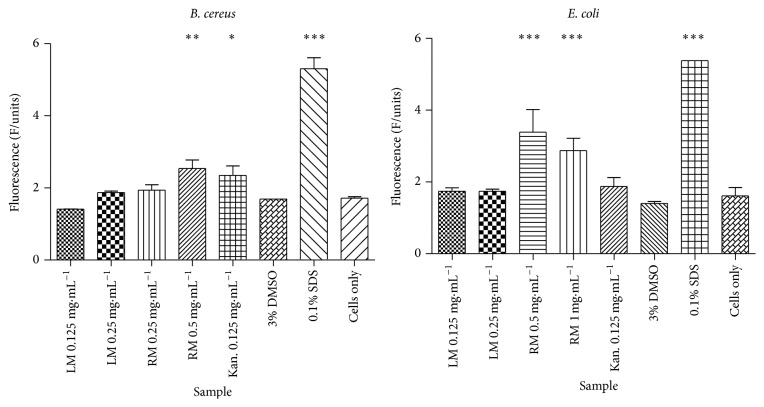
Fluorescence of nucleic acid binding propidium iodide after exposure of* B. cereus* and* E. coli* to* T. welwitschii* plant extracts. The* T. welwitschii* extracts were tested at the MIC and 2-fold the MIC, while kanamycin, 3% DMSO, 0.1% SDS, and untreated cells were used as controls. *n* = 2. The test was conducted twice. The test for significance was carried out by comparing all samples to cells only. ^*∗*^ *P* < 0.05. ^*∗∗*^ *P* < 0.01. ^*∗∗∗*^ *P* < 0.001. LM: leaf methanol; RM: root methanol; Kan.: kanamycin; DMSO: dimethyl sulfoxide; SDS: sodium dodecyl sulphate.

The fluorescence of propidium iodide in* B. cereus* and* E. coli* cells after exposure to the* T. welwitschii* leaf methanol and root methanol extracts is shown in Figure 2. Against* B. cereus*, the fluorescence of propidium iodide increased as the concentration of the root methanol extract increased. The root methanol extract caused the most nucleic acids leakage. At the MIC (0.25 mg/mL) and double the MIC (0.5 mg/mL), the fluorescence of propidium iodide was 1.94 fluorescence units (F/units) and 2.53 F/units, respectively. The fluorescence at 0.5 mg/mL was significantly higher than the fluorescence of propidium iodide in the untreated* B. cereus* cells, which was 1.72 F/units.

In* E. coli*, following exposure to the leaf methanol extract, the fluorescence of propidium iodide was 1.741 F/units and 1.743 F/units after exposure to the MIC (0.125 mg/mL) and double the MIC (0.25 mg/mL), respectively. The root methanol extract at 0.5 mg/mL and 1 mg/mL (MIC and double the MIC, resp.) caused nucleic acid leakage in* E. coli* cells. However, as the concentration of plant extracts increased, the fluorescence of propidium iodide decreased: 3.386 F/units at the MIC and 2.871 F/units at 2-fold the MIC. Untreated* E. coli* cells had a propidium iodide fluorescence of 1.61 F/units. SDS was able to cause nucleic acid leakage in* B. cereus* and* E. coli*. In* B. cereus* the fluorescence of propidium iodide in cells exposed to DMSO was 1.692 F/units which was similar to that in untreated cells. The same was true for* E. coli* cells, where the fluorescence of propidium iodide was 1.403 F/units for cells treated with DMSO.

### 3.5. Determination of the Effect of* T. welwitschii* Extracts on Protein Leakage

**Figure 3:**
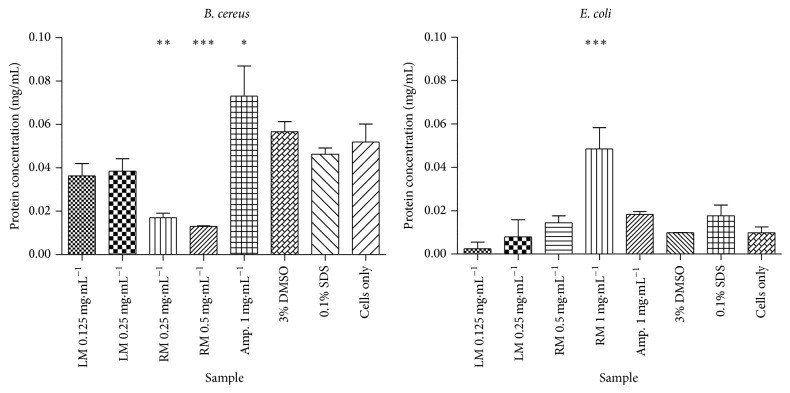
Graph of protein leakage in* B. cereus* and* E. coli* after exposure to the* T. welwitschii* leaf and root methanol extracts. The protein concentration is shown in mg/mL. *n* = 2. The test was conducted three times. The test for significance was carried out by comparing all samples to cells only. ^*∗*^ *P* < 0.05. ^*∗∗*^ *P* < 0.01. ^*∗∗∗*^ *P* < 0.001. LM: leaf methanol; RM: root methanol; Amp.: ampicillin; DMSO: dimethyl sulfoxide; SDS: sodium dodecyl sulphate.

In* B. cereus* cells the* T. welwitschii* extracts do not cause protein leakage (Figure 3). In the presence of the plant extracts, less protein is lost compared to untreated cells. The root methanol extract at 0.25 mg/mL and 0.5 mg/mL (the MIC and double the MIC, resp.) caused significantly less protein leakage than the untreated cells. Ampicillin caused the leakage of large amounts of protein (0.073 mg/mL). In the cells treated with SDS, 0.46 mg/mL of protein was leaked, while in those treated with DMSO 0.057 mg/mL of protein was leaked out. These amounts were similar to the untreated cells: 0.052 mg/mL of protein was leaked from the cells.

In* E. coli* (Figure 3), the root methanol extract caused protein leakage at 1 mg/mL (double the MIC): 0.048 mg/mL. The protein leakage observed in the untreated cells was 0.0098 mg/mL. DMSO did not change the amount of protein leakage in* B. cereus* cells.

## 4. Discussion

The search for new antimicrobial agents has been necessitated by the increase in antimicrobial resistance in recent years. Fifty thousand people die every day worldwide due to infectious diseases [33]. Natural plant products are a good source of new antimicrobials as they generally have low toxicity, cause minimal environmental pollution, have a low risk of development of resistance by pathogens [34], are cheap, and are generally safer than synthetic medicines [35]. The use of known medicinal plants is advantageous as they have been prescreened over thousands of years, resulting in a higher probability of isolating useful and safe compounds from them than from plants not in use by humans already [36]. Plant materials are either present in or have provided models for approximately 50% of drugs [35]. Screening* T. welwitschii* and* C. welwitschii* for antibacterial activity was more likely to be successful as these plants are already used for medicinal purposes.

The extracts from the two plants were generally more effective against* B. cereus* than* E. coli*.* B. cereus* is a Gram-positive bacterium while* E. coli* is Gram-negative, suggesting that the plant extracts are more effective against Gram-positive than Gram-negative bacteria. According to the literature, plant extracts are generally more active against Gram-positive than Gram-negative bacteria [37]. Gram-negative bacteria are more resistant to antibacterial agents than Gram-positive bacteria [38, 39] because of the presence of the outer membrane that acts as a permeability barrier [38]. Seasotiya and Dalal [40] and Narayan [38] found that Gram-positive bacteria were more susceptible to the tested plant extracts than Gram-negative bacteria.

The MBC test results showed that when exposed to 4 mg/mL of* C. welwitschii* root methanol extract,* E. coli* grew fairly well, but at 2 mg/mL the extract killed the bacteria. The anomalous result observed may have been caused by high levels of nutrients present in the tuber that allowed the bacteria to overcome any bactericidal substances in the root extract. Nutrients would be more concentrated at 4 mg/mL compared to lower concentrations.

All of the plant extracts except the* T. welwitschii* root water extract against* E. coli* were able to inhibit the growth of the bacteria. Many studies have shown that plant extracts have antibacterial activity. Plants found to have antibacterial activity include* Leonotis nepetifolia* [38],* Melia azedarach* [21],* Avicenna marina* [35],* L. erythrorhizon* [19],* Adansonia digitata*,* Tamarindus indica*,* Aframomum alboviolaceum,* and* Ocimum gratissimum* [41].

The plant extracts had much higher MICs and MBCs than those produced by the antibiotics against the bacteria, showing that they are not as effective as the antibiotics. The use of whole extracts as opposed to purified compounds may be responsible. There may have been a lack of specificity as there were many compounds working together at reduced concentrations. Interactions between these compounds may also reduce the effectiveness of the plant extract. Studies have shown that plant extracts, though effective against bacteria, are in most cases not as effective as antibiotics [9, 36, 42–44].

The presence of efflux pumps on the bacterial membrane causes resistance in bacteria [45]. Bacteria use these efflux pumps to expel antibiotics from the cell until their concentration is too low to be effective against the bacteria [46]. The extracts from* T. welwitschii* caused accumulation of more R6G in* E. coli* cells than in* B. cereus* cells.* T. welwitschii* was more capable of binding to the efflux pumps on* E. coli* than those on* B. cereus*. However,* T. welwitschii* failed to cause significant drug accumulation in both bacteria.

*C. welwitschii* in* B. cereus* was ineffective as an efflux pump inhibitor. The* C. welwitschii* leaf methanol extract was a more effective efflux pump inhibitor in* E. coli* than reserpine. The* C. welwitschii* leaf methanol extract has potential for use as an efflux pump inhibitor. R6G was expected to accumulate in the bacterial cells in the absence of glucose while efflux of R6G would occur in the cells exposed to glucose. R6G may not have accumulated without glucose as cells may use proton driven antiporters and/or ATP driven (ATP-binding cassette (ABC)) transporters to expel drugs [12]. The R6G may have been expelled from the cells through the proton driven antiporters as opposed to the ABC transporters.* E. coli* has 19 antiporters belonging to the major facilitator superfamily, small multidrug resistance family and the resistance/nodulation/cell division family [12], and 80 ABC transporter proteins [47]. Proton driven antiporters expel drugs by coupling drug efflux to the influx of a proton, H^+^, while ABC transporters couple drug efflux to the hydrolysis of ATP [12]. The AcrAB-TolC pump is a member of the resistance-nodulation-cell division (RND) family of tripartite multidrug efflux pumps ubiquitous throughout Gram-negative bacteria [48]. In* E. coli*, the multidrug efflux pump has been shown to expel a wide range of antibacterial agents [48].

Many plant extracts with the ability to promote drug accumulation in bacterial cells have been studied.* Artemisia absinthium* has been found to contain efflux pump inhibitory compounds [11].* Hydrastis canadensis*,* Curcuma longa*,* Capsicum annum,* and* Elettaria cardamomum* have been found to inhibit efflux pump activity [40, 49].

Some compounds are capable of disrupting the integrity of the bacterial cell wall/membrane; for example, polymyxins increase bacterial membrane permeability and lipopeptides like daptomycin and crystallomycin bind to bacteria and cause rapid depolarisation of the bacterial membrane and eventual cell death [50]. The* T. welwitschii* root methanol extract was capable of causing damage to the* B. cereus* and* E. coli* membranes resulting in nucleic acid leakage (Figure 2). DMSO did not cause damage to the bacterial membranes as the protein and nucleic acid leakage from the bacterial cells did not change significantly when the cells were exposed to DMSO. Although the root methanol extract caused leakage of nucleic acids in* E. coli*, the leakage decreased with increasing concentration of plant extracts. Further studies are needed to determine the cause of this phenomenon.* Cocos nucifera *husk extracts caused leakage of nucleic acids in bacteria [51].* Plumbago zeylonica*,* Leucas aspera,* and* Hemidesmus indicus* were all found to be capable of causing nucleic acid leakage in bacteria [52]. The plant extracts from these plants were found to contain phenols and flavonoids, resulting in the authors concluding that the membrane disrupting activities of the extracts may be due to the activities of these phytochemicals [52].

Ampicillin was able to cause significant protein leakage in* B. cereus* but not in* E. coli*. Ampicillin inhibits bacterial cell wall synthesis [1]. Ampicillin is thus expected to be more effective against Gram-positive bacteria than Gram-negative bacteria due to the differences in their structures. Gram-positive bacteria have thick cell walls (20–80 nm) composed mainly of peptidoglycan while Gram-negative bacteria have thin, inner peptidoglycan layers (2–7 nm) and an outer membrane (7-8 nm) of lipid, protein, and lipopolysaccharide [1].

Protein leakage was only observed in* E. coli* after treatment with 1 mg/mL* T. welwitschii* root methanol extract. The root methanol extract was able to bind to and disrupt the* E. coli* membrane and not the* B. cereus* cell wall. Some authors have found that plant extracts are capable of causing membrane disruption [53, 54]. Henie et al. [53] found that* Psidium guajava* was capable of causing protein leakage in various bacteria, while Akinpelu et al. [55] found that extracts from* Garcinia kola* caused protein leakage in bacteria, including* E. coli*.

## 5. Conclusions

In conclusion, the leaf and root extracts of* C. welwitschii* and* T. welwitschii* were shown to have antibacterial activity against* B. cereus* and* E. coli*.* T. welwitschii* extracts were more potent growth inhibitory activity against the bacteria than the* C. welwitschii* extracts. Further analysis is needed to investigate the exact mode of activity of the plant extracts and the* in vivo* toxicity of the plant extracts and to determine the phytoconstituents present in the plant extracts.* T. welwitschii* may be a potential source of antibacterial agents.

## Figures and Tables

**Table 1:** Zones of inhibition produced after exposure of bacteria to plant extracts and antibiotics. The zone of inhibition refers to the diameter of the circle in which no growth of cells was observed, minus the diameter of the filter paper disk (6 mm). The assay was conducted twice.

Plant extract/antibiotic	*B. cereus* zone of inhibition (mm)	*E. coli* zone of inhibition (mm)
*C. welwitschii* leaf methanol	2.5 ± 0.6	1.3 ± 0.5
*C. welwitschii* leaf water	0	0
*C. welwitschii* root methanol	0.8 ± 0.29	0.5 ± 0.0
*T. welwitschii* leaf methanol	3.3 ± 0.5	2.3 ± 0.5
*T. welwitschii *leaf water	0	0
*T. welwitschii *root methanol	6 ± 0	4.5 ± 1.2
*T. welwitschii* root water	1.8 ± 0.5	2 ± 0.6
Ampicillin	9 ± 0.0	12 ± 0.8
Methanol	0	0
Distilled water	0	0

**Table 2:** MICs and MBCs of plant extracts and antibiotics against *B. cereus* and *E. coli*. The assay was conducted twice.

Bacterium	Extract/antibiotic	MIC (mg/mL)	MBC (mg/mL)
*B. cereus*	*C. welwitschii* leaf methanol	2	>4
	*C. welwitschii *root methanol	2	>4
	*T. welwitschii *leaf methanol	0.125	>0.5
	*T. welwitschii *root methanol	0.25	>1
	*T. welwitschii *root water	0.5	>2
	Ampicillin	0.004	0.008
	Kanamycin	0.0005	0.004
	Norfloxacin	0.00025	0.002

*E. coli*	*C. welwitschii* leaf methanol	0.5	2
	*C. welwitschii *root methanol	1	a
	*T. welwitschii *leaf methanol	0.125	>0.5
	*T. welwitschii *root methanol	0.5	2
	*T. welwitschii *root water	>4	>4
	Ampicillin	0.008	0.016
	Kanamycin	0.001	0.008
	Norfloxacin	0.00025	0.004

The > indicates that the MIC and MBC were higher than the tested concentrations.

^a^At 4 mg/mL of the *C. welwitschii *root methanol extract, *E. coli *cells were able to grow, but they were unable to grow at 2 mg/mL.
